# Higher sowing density of pearl millet increases productivity and water use efficiency in high evaporative demand seasons

**DOI:** 10.3389/fpls.2022.1035181

**Published:** 2022-12-08

**Authors:** Raphaël Pilloni, Aliou Faye, Aparna Kakkera, Jana Kholova, Romiel Badji, Coumba Faye, Vincent Vadez

**Affiliations:** ^1^ Diversité, adaptation, développement des plantes (DIADE), University of Montpellier, Institut de Recherche pour le Développement, Montpellier, France; ^2^ Centre d’étude régional pour l’adaptation des plantes à la sècheresse, Thiès, Senegal; ^3^ International Crop Research Institute in Semi-Arid Tropics, Hyderabad, India; ^4^ Department of Information Technologies, Faculty of Economics and Management, Czech University of Life Sciences Prague, Prague, Czechia; ^5^ Laboratoire Mixte International Adaptations des Plantes et micro-organismes associés aux Stress Environnementaux (LAPSE), Dakar, Senegal

**Keywords:** climate change, sustainable intensification, canopy architecture, semi-arid tropics, vapor pressure deficit

## Abstract

**Introduction:**

Pearlmillet is themain subsistence crop for smallholder farmers systemswhere it is grown at low plant density. Intensifying pearl millet cultivation could boost productivity although it may have trade-offs. Increasing planting density would indeed increase the leaf area and the related water budget, whereas a denser canopy could create a more favorable canopymicroclimate to the benefit of the water use efficiency (WUE) of the crops. The first aim of this work was to test the yield response of popular pearlmillet varieties to an increased density and to assess possible genotypic variation in this response. The second aim was to measure the water use and the WUE of the crop in different densities.

**Method:**

To this end we designed several field and lysimetric experiments To increase the robustness of the results, these trials were carried out in India and Senegal, using two independent sets of genotypes adapted to both sites.

**Results:**

In the field, the higher sowing density significantly increased yield in all genotypes when trials were carried out in high evaporative demand conditions. There was no genotype x density interaction in these trials, suggesting no genotypic variation in the response to density increase. The high-density treatment also decreased the vapor pressure deficit (VPD) in the canopies, both in the field and in the lysimeter experiments. In the lysimeter trials, although the higher density treatment increased water use, the resulting increase in biomass was proportionally higher, hence increasingWUE of the crops in all genotypes under high density. The increase in yield under high density was closely related to the increase in WUE, although this link was more tight in the high- than in the low evaporative demand seasons. This confirmed a strong environmental effect on the response to density of all genotypes tested.

**Discussion:**

Although they did not open a scope for breeding density tolerant cultivars, these results highlight the possibility to improve pearl millet yield by increasing the density, targeting specifically areas facing high evaporative demand.

## Introduction

Pearl millet is a sustainable crop for the semi-arid tropics regions (SAT) where it is grown. In the Sahel region, it is a major staple food and represents the main economic supply for smallholder farmers. Around 45% of the world production of pearl millet takes place in Western and Central Africa (WCA) (FAO, 2017). In India, pearl millet is the fourth most produced cereal and therefore it also represents a major agronomic interest in this densely populated region (ICAR, 2020). In these small-farming areas, pearl millet is traditionally sown under low to very low density (Bationo et al., 1990). In other crops like maize, there has been a progressive increase in sowing density that has contributed to the yield increase of this crops over the years, although this has never been a specific breeding target (Mansfield & Mumm, 2014; York et al., 2015; Di Matteo et al., 2016). In our knowledge, no similar increase in the density of sowing of pearl millet has taken place, although a sustainable intensification of this crop is needed to cater for the increased demand of its grain and fodder, especially in the current tense situation of world cereal markets. Increasing the traditional sowing density could be a lever to increasing pearl millet productivity. Therefore, one hypothesis of this work is that there is room to increase the productivity of pearl millet and there may be genotypic variation is the degree of response to increased sowing density, which could open a breeding target.

One issue with crop intensification in Sahelian climates is water scarcity. An expected increase in the leaf area index (LAI) caused by an increased sowing density would normally increase plant water requirement. This needs to be assessed and this is one objective of this work. In addition, the vapor pressure deficit (VPD) is high in those climates and causes an atmospheric drought that will become even more acute in future climate (Kholová et al., 2010; Kholová et al., 2012; Asseng et al., 2015). This must also be dealt with. There, an increase in density of planting could bring a benefit. Indeed, the increase in leaf area index could create a microclimate within the canopy with a milder VPD, as shown recently in sorghum (Pilloni et al., 2022, under review). This may mechanically increase the water use efficiency (WUE) of the crops (Fletcher et al., 2008; Grossiord et al., 2020) a trait of agronomic importance. Water use efficiency is indeed inversely related to the VPD that the plants are exposed to (Thomas R. Sinclair et al., 1984; Sinclair et al., 2005; Vadez et al., 2014), and then is higher at lower VPD’s. Our hypothesis is that an increase in sowing density would also increase the WUE of denser canopies, and then partially compensate for the increased water demand from higher LAI.

The objectives of this study were then to test whether pearl millet productivity could be increased by increasing its sowing density, whether there was genetic variation in this response, and how much this change in crop management could affect water use and WUE of the crops. To that end, we conducted several field and lysimeter experiments to quantify yield, water use and WUE in different cultivars and densities conditions. To increase the robustness of the results, our hypotheses were tested in two different locations, i.e. at the ICRISAT site in Patancheru in India and at the CNRA station in Bambey in Senegal, with two distinct panels of released cultivars adapted to both sites.

## Material and methods

### Locations and biological material

**Figure 1:**
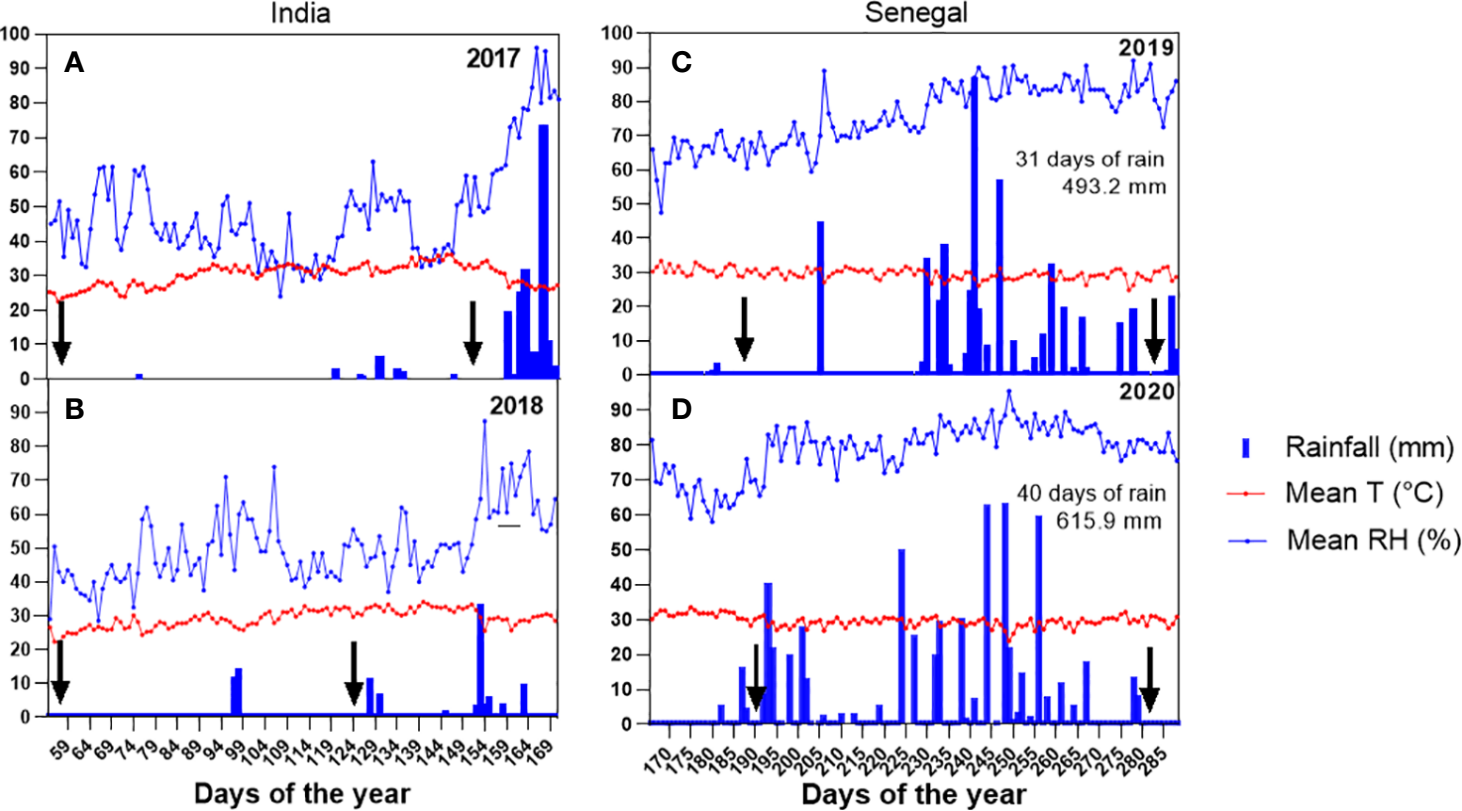
Mean temperature, mean relative humidity, and rainfall recorded at the ICRISAT station **(A, B)** and at the Bambey **(C, D)** meteorological station during the field trials. The arrows in each panel correspond to the sowing and harvest dates in each crop cycle.

Field and lysimeter trials were conducted during the 2017 and 2018 dry seasons and during the 2018 rainy season in India at the ICRISAT research station (Hyderabad, 17°31’01.3”N 78°16’33.4”E) and during the 2019 and 2020 rainy seasons and 2021 dry seasons in Senegal at the CNRA research station (Bambey, 14°42’ N; 16° 28’ W). All trials in India, both in the field and in the lysimeters, were fully irrigated trials. The lysimeter trial in Senegal was also fully irrigated. The field trials in Senegal were rainfed but received either supplemental irrigation at sowing or abundant rainfall to ensure at least 60mm of water were received every week. Therefore, no water stress was applied in any of these experiments. Soils at the ICRISAT farm are Alfisol while those at the Bambey farm are typical Sahelian sandy soil with more than 90% sand. The biological material used in this study consisted in two panels of genotypes. Panel 1 included 20 commercial elite varieties from the ICRISAT-India breeding program and from private companies, and was tested in India. Panel 2 included 30 elite varieties from the ICRISAT breeding program for West Africa along with regional elite lines, and was tested in Senegal. Temperature, hygrometry, and rainfall was recorded every day in both ICRISAT and CNRA stations and used to characterize the seasons of experiment (**Figure 1**).

### Yield assessment in the field

In India, two field trials were carried out in the 2017 and 2018 summer season (February – May) characterized by high VPD. The field was mechanically tilled and 100 kg/ha DAP was applied before sowing and 100 kg/ha urea applied 15 days after sowing. The 20 genotypes from the panel 1 were manually sown in 8m² (4m long, 2m wide) plots of two different densities. A low-density treatment (LD) consisted in plot of 4 rows spaced at 60 cm intervals from each other and 15 cm between plants in the same row, leading to a density of 12 plants/m². High-density treatment consisted in plots of 8 rows spaced with 30 cm intervals from each other and 15 cm between plants within the row and leading to a density of 24 plants/m² (**Supplementary figure 1A**). The field trials were fully irrigated and received 40 mm of irrigation every week from sowing to maturity. These trials followed a fallow period during the previous rainy season. Grain and vegetative biomass yield were harvested from the entire micro plot in 2017. A storm in 2018 forced us to harvest soon after flowering, at the very beginning of grain filling. We measured total aboveground biomass in this case. Samples were dried 72h in the oven.

In Senegal, field trials were carried out during the 2019 and 2020 rainy seasons (June-September). The trials were rainfed but received supplemental irrigation if needed to ensure 60mm water every week to the crop. The field was mechanically tilled and 150 kg/ha DAP (15-15-15) was applied 10 days after sowing and 100 kg/ha urea applied 15 days after sowing. The 30 genotypes from the panel 2 were manually sown in three different densities. A plot consisted in 3.25 m² (1.8m long, 1.8 wide). The standard density treatment (D1) consisted of lines of pockets, each thinned to three pearl millet plants, separated from each other by 90 cm in the row and between rows (3.2 plants/m²). Two increased density treatments were set up, consisting in adding either one pocket of three plants within the row (D2, 6.4 plants/m²), or both within in the row and between the rows (D3, 12.8 plants/m²) (**Supplementary figure 1B**). At grain maturity, panicles were harvested, sun dried and weighed to measure grain yield.

In both sites, the experimental design was a completely randomized block design, with density treatment as the main block and genotypes randomized three and four times in each main block in Senegal and India respectively.

### Measurement of the leaf area index

The leaf area index (LAI) was measured in the 2018 field trial in India and in the 2020 field trial in Senegal, using a 1-meter long ceptometer (AccuPAR LP-80, Meter in India and Sun scan type SS1, Delta-T devices in Senegal) (Francone et al., 2014). The aim was to compare the soil coverage allowed by the higher density, putative genotypic differences in LAI, and a possible link between the LAI and the genotypic response to an increased density. In both sites, LAI measurements were made at two dates, around and after canopy closure. In each plot, two measurements were taken from above the plants to assess the incident radiation at that particular time. The device was then placed diagonally on the ground, between two (Senegal) or four (India) rows according to the treatment and site, and four light measurements were done in each plot, at different locations of the plot. The ceptometer measured the amount of photosynthetically active photon in µmol.s^-1^.m² ^-1^ and converted the light quantity into a leaf area index using the following formula: $LAI=\ln\left(\frac{I}{Io}\right)/k$ where *I* is the incident light above the canopy Io, the light at ground level and *k* a crop extinction equal to 0.6 for pearl millet crops. Measurement was done at 33 and 40 days after sowing (DAS) in India and at 43 and 63 DAS in Senegal, close to sun zenith time.

### Soil core to measure water content

Water content of soil cores were measured at harvest to assess how the different density treatments had affected water content in the different layers of the soil profiles. Three and four micro plots in India and Senegal, respectively, were randomly selected in the different density treatments. Soil cores of 90 cm depth were taken in between two rows using a manual auger. Each core was separated in layers of 30 cm and immediately weighed. The soil was then stored in metal boxes and put in the oven for 72h for complete drying. The samples were then re-weighed. The ratio of fresh weight to dry weight allowed the calculation of the water content (percentage) of the samples for each soil horizon studied using the following formula: $\text{soil moisture}\left(\%\right)=\frac{\left(P_{H}-P_{S}\right)}{P_{S}}*100$ as proposed by Anderson & Ingram (1993).

### Water use and WUE measurements in lysimeter platforms

Three trials were carried out in both Senegal and India on similar lysimetric platforms. The platforms consisted of PVC tubes installed side by side in long trenches. The tubes were filled with soil from the fields adjacent to the platforms (Alfisol in India, sandy soil in Senegal) (see V. Vadez et al., 2011)for more details about the process). A pulley system associated with an S-type load cell (Mettler-Toledo, Geneva, Switzerland) allowed to weigh regularly each tube individually and to measure the water use of each plant through mass differences between consecutive weighing. A replication consisted in a set of four tubes, all being planted with one plant each in the high density treatment, whereas only two tubes were planted in the low density while the two others remained empty (**Supplementary figure 2**). The empty tubes’ water losses were measured and were integrated in the total water use of the replication. The cylinders were brought to field capacity before sowing by watering cylinders abundantly and letting them drain. After sowing, cylinders received 500mL on alternate days. Before starting the weighing, cylinders were again brought to field capacity, and the field capacity weight was taken as a benchmark for re-watering. At each subsequent weighing, water was added to each cylinder to reach field capacity weight minus a buffer of 2 kg to avoid possible drainage. At harvest, total plant water use was then the sum of weight difference between the last weight and the initial field capacity weight plus water added during the period of weighing. The plants were harvested after a period of growth of 7 to 9 weeks according to the trials and total aboveground biomass was measured after drying for three days in an oven at 70°C. The water use efficiency (WUE) was calculated by dividing the total biomass produced by the total water used, and expressed in g biomass per liter of water used by the crop (g L^-1^). The experimental design was a completely randomized block design, with density treatment as the main block and genotypes randomized three and four times in each main block in Senegal and India respectively. Panel 1 was tested in two seasons contrasting for the evaporative demand (i.e. dry and rainy season 2018) in India. Panel 2 was tested during the 2021 post rainy season in Senegal (characterized by a high evaporative demand).

### Canopies vapor pressure deficit assessment

Vapor pressure deficit (VPD) in the canopies of different density was measured with temperature and relative humidity sensors (TinyTag ultra 2, TGU-4500, Gemini Datalogger Ltd, Chichester, UK), during 15 days of the 2018 high VPD season lysimeter trial in India and 6 days during the 2020 low VPD season field trial in Senegal. In India, the sensors were installed at 53 days after sowing (DAS) (corresponding to flowering time in most of the genotypes) and at 89 DAS in Senegal, corresponding to the last week before grain maturity. Data was recorded every 30 min from 7am to 7pm. Daily average of the VPD was calculated according the following formula:

VPD = (100 - %RH x SVP/1000) where SVP (saturated vapor pressure) is 610.7 x 10**^(7.5 x T°C)/(273.5+T°C)^**.

### Statistical analysis

The statistical analysis (Analysis of variance, t-test, simple linear regressions) presented in this study was performed using GraphPad Prism (version 9.4.1 for Windows, GraphPad Software, San Diego, California USA, www.graphpad.com) and in R software.

## Results

### Similar dry seasons in India but contrasting rainy seasons in Senegal

The 2017 and 2018 dry seasons field trials in India were carried out under fairly similar conditions, i.e. characterized by high temperature and low relative humidity with no or very limited rainfall (3 rainfall days summing 27mm in 2017 and 7 rainfall days summing 17mm and 2018, **Figure 1A, B**). By contrast, the two rainy seasons field trials in Senegal were carried out under different rainfall amount and distribution during the season. In 2019, it rained on 35 days during the growing season, for a total of 493 mm (**Figure 1C**). By contrast, during the 2020 rainy season, it rained on 42 days for a total rainfall of 643 mm (**Figure 1D**). Specifically, during the first 60 days of the crop cycle (before flowering), 2 rainy days only occurred in 2019 against 14 in 2020 over the same period. (**Figure 1C, D**). Relative humidity was also lower by 5% on average of the season and 7% on the 60 first days of the crop cycle in 2019 compared to 2020. Light intensity, collected in a nearby weather station 25km apart, was also 10% higher in 2019 than in 2020 during the initial 60 days of the crop (data not shown). The average of daily VPD in the two lysimeter trials carried out in 2018 in India (summer and rainy seasons), differed and were 2.1 kPa and 0.91kPa during the dry and rainy season respectively (detailed data not shown). The average of daily VPD in the lysimeter trials carried out in the dry in Senegal was 1.9 kPa

### High-density increases the yield differently across years and locations

**Figure 2:**
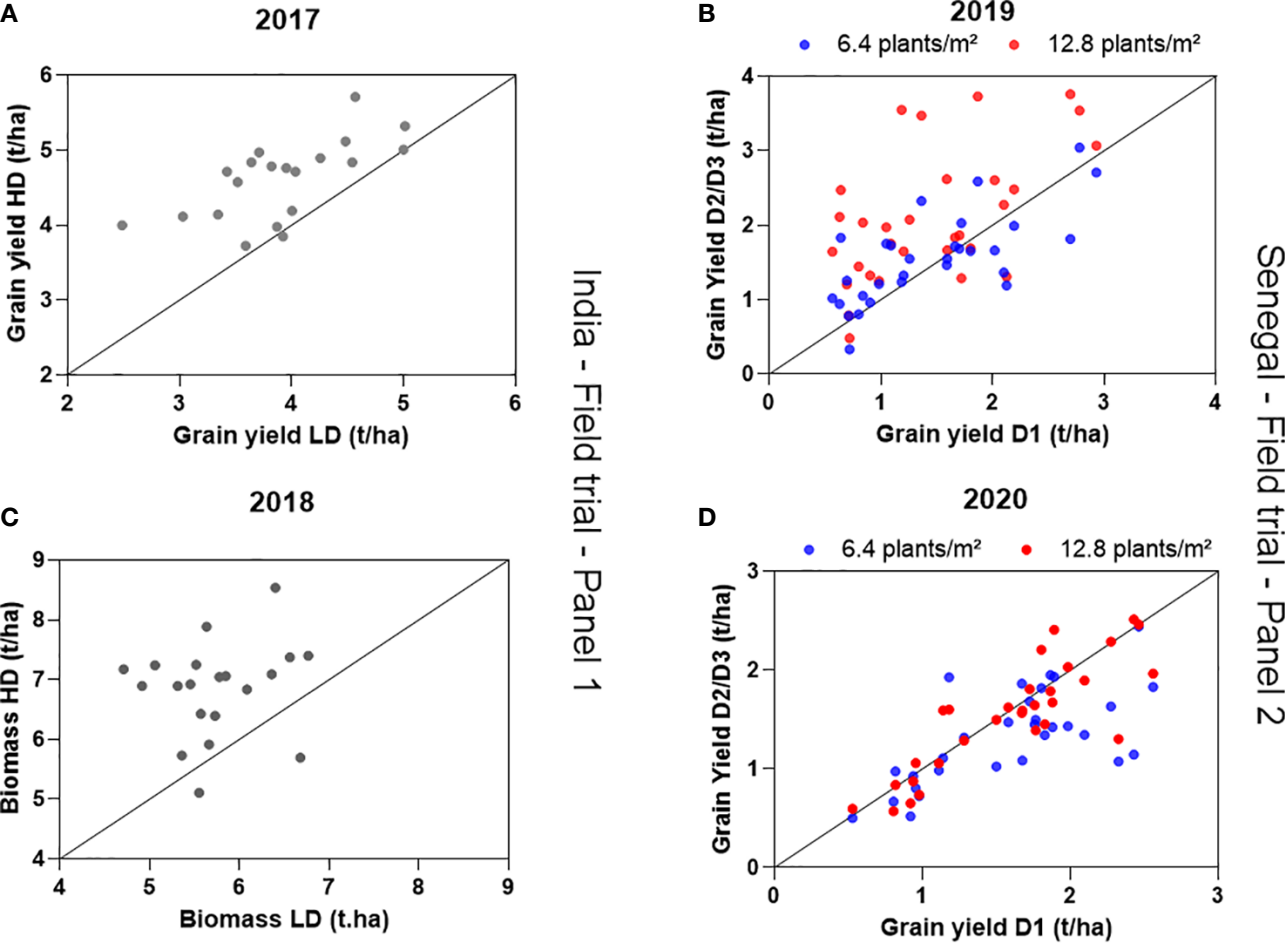
Grain yield under high density (HD) as a function of grain yield under low density (LD) in 2017 **(A)**, total aboveground biomass under high density (HD) as a function of total aboveground biomass under low density (LD) in 2018 **(B)** in India **(A, B)**. Grain yield under medium and high density (D2 and D3) as a function of grain yield under low density (D1, 3.2 plants/m^2^)) in 2019 **(C)** and 2020 **(D)** in Senegal. Data are means of 4 replicated plots in India and 3 replicated plots in Senegal, for each genotype-by-density combination.

The high-density treatment increased yield significantly in the two field trials carried out in India. The average grain yield for the 2017 trial was 3.41 ± 0.42 t/ha in low-density and 3.92 ± 0.46 t/ha. (**Figure 2A**). A two-way ANOVA highlighted a strong density effect on yield (Wald statistic, p-value=0.0001), a significant genotypic effect but no genotype-by-density interaction, indicating that no genotype responded better to the high-density treatment than another. The same trend was observed during the 2018 trial where the total aboveground biomass (vegetative biomass + emerging panicles) was used as a proxy for the yield response. There, aboveground biomass had an average of 5.74 ± 0.79 t/ha under low-density and 6.84 ± 0.58 t/ha under high density (**Figure 2B**). Again, a strong density effect was highlighted (Wald statistic, p-value=0.0001), with a significant genotypic effect, and again no genotype-by-density interaction. Genotypic means of yield in both high and low density treatments are presented in the **Supplementary Table 1**.

In Senegal, the response of the panel of genotypes tested was drastically different across the two years, showing a positive yield response to density in 2019, and a slightly negative one in 2020. During the 2019 field trial, the grain yield significantly increased with density and was 1.45 ± 0.68 t/ha in the lowest density D1, 1.55± 0.60t/ha in the intermediate density D2, and 2.10± 0.88 t/ha in the highest density D3 (**Figure 2C**). The ANOVA analysis revealed a strong genotypic effect, explaining most of the variation, although the density effect was also highly significant (ANOVA, Wald statistic, **Table 1**). No genotype × density interaction was found in the three-density treatment dataset, although a slight but significant genotype x density interaction effect (p-value = 0.01) was found in a two-way ANOVA that was ran with D1 and D3 data from 2019. During the 2020 trial, the grain yield significantly decreased in the highest density treatment. Average yield data were the following in the three density conditions: 1.6 ± 0.55 t/ha in D1, 1.53 ± 0.56 t/ha in D2 and 1.3 ± 0.47 in D3. Analysis of variance on yield showed a genotypic effect (p-value<0.0001) and a slight density effect (p-value= 0.046) with no genotype-by-density interaction effect (**Figure 2D**). Genotypic means of yield in the three density treatments are presented in the **Supplementary Table 2**.

**Table 1:** Two way ANOVA table showing significance and Wald statistic for the yield obtained in two different density (HD, LD) in India in 2017 and 2018 and in the three different density (D1, D2, D3) in 2019 and 2020 in Senegal.

Source of Variation	Yield 2017 (India)		Yield 2018 (India)	
Two-way ANOVA	Significance	Wald statistic	Significance	Wald statistic
Genotype	***	2.95	ns	1.33
Density	****	26.7	****	29.36
Genotype x Density	ns	0.97	ns	1.01
**Source of Variation**	**Yield 2019 (Senegal)**		**Yield 2020 (Senegal)**	
Two-way ANOVA	Significance	Wald statistic	Significance	Wald statistic
Genotype	****	7.8	****	4.02
Density	****	23.9	*	3.13
Genotype x Density	ns	1.3	ns	0.36

*p-value < 0.05, ***p-value < 0.001, ****p-value < 0.0001.

ns, non-significant.

### High-density increases LAI

**Figure 3:**
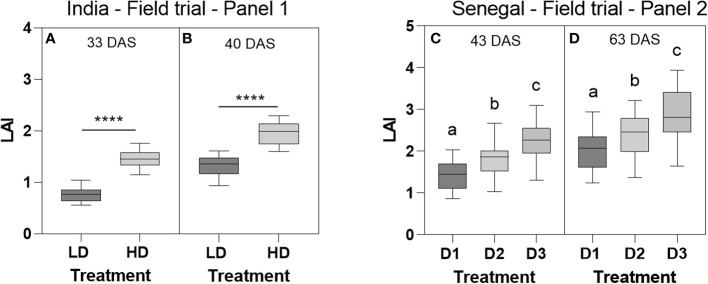
Leaf area index (LAI) measured at 33 DAS **(A)** and 40 DAS **(B)** in the 2018 field trial in India under low density (LD) and high density (HD), and at 43 DAS **(C)** and 63 DAS **(D)** in the 2020 field trial in Senegal in the low (D1, 3.2 plants/m^2^), intermediate (D2, 6.4 plants/m^2^), and high (D3, 12.8 plants/m^2^) density treatments. Data are means and standard deviation of 20 genotypic means in India and 30 genotypic means in Senegal. Genotypic mean values were the average of 4 replicated plots in India and 3 replicated plots in Senegal, for each genotype-by-density combination. ****, p-value <0.0001.

In the different field trials, the leaf area index measured at two different times after sowing was always larger under higher density for all genotypes. A two-way ANOVA showed a significant density effect but no genotype or genotype-by-density interaction effect in the two dates of measurement (**Figure 3A, B**).

In the 2020 field trial in Senegal, LAI measurement made at 43 DAS showed significant differences between different densities and genotypes while no genotype-by-density interaction was found. Strong and significant density and genotypic effect was found for LAI for the two dates of measurement (ANOVA, p-value<0.0001, Tuckey’s multiple comparisons test) but no genotype-by-density interaction (**Figure 3C, D**). Genotypic values of LAI measured at the different dates, in the different trials were plotted against the yield, and did not show any significant relation (data not shown).

### Denser canopies have lower VPD

**Figure 4:**
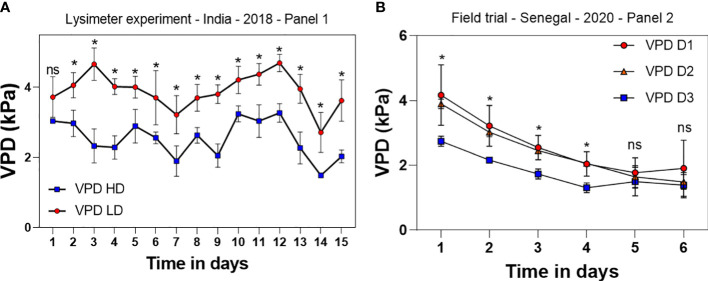
Vapor pressure deficit (VPD) measured on a 15 days period during vegetative stage in high density (HD) and low density (LD) canopies in the lysimeter trial in India in the germplasm panel 1 during the dry season **(A)** and in the 2020 field trial in the three different densities (low (D1, 3.2 plants/m^2^), intermediate (D2, 6.4 plants/m^2^), and high (D3, 12.8 plants/m^2^)) on a 6 days period before harvest in Senegal **(B)** in the germplasm panel 2. Each data point is the average of data collected in three plots for each of the densities. ns, non-significant. *p-value < 0.05.

In both the lysimeter and the field conditions, the high-density treatment reduced significantly the VPD within the canopies. Mean VPD during the 15 days of recording in India was 2.86 kPa in the HD treatment and 3.82 kPa in the LD treatment, i.e. a 0.96 kPa difference on average (paired t-test, p-value<0.0001) (**Figure 4A**). In Senegal during the 2020 rainy season, VPD in the highest density treatment D3 was significantly lower (average VPD of 1.98 kPa) than in the lowest density treatment D1 (average VPD of 2.99 kPa in the first 4 days of the measurement) (paired t-test, p-value<0.01) (**Figure 4B**). VPD differences were not significant between the two lowest densities D1 and D2 (Paired t-test, p-value = 0.11). On the days 5 and 6, ambient VPD dramatically decreased because of rainfall (2.02 kPa on day 5 and 1.76 kPa on day 6), leading to the loss of the density effect on the within-canopy VPD as non-significant differences were recorded for those two particular days, regardless of the treatments compared (**Figure 4B**).

### Higher density increased water use efficiency in all genotypes

**Figure 5:**
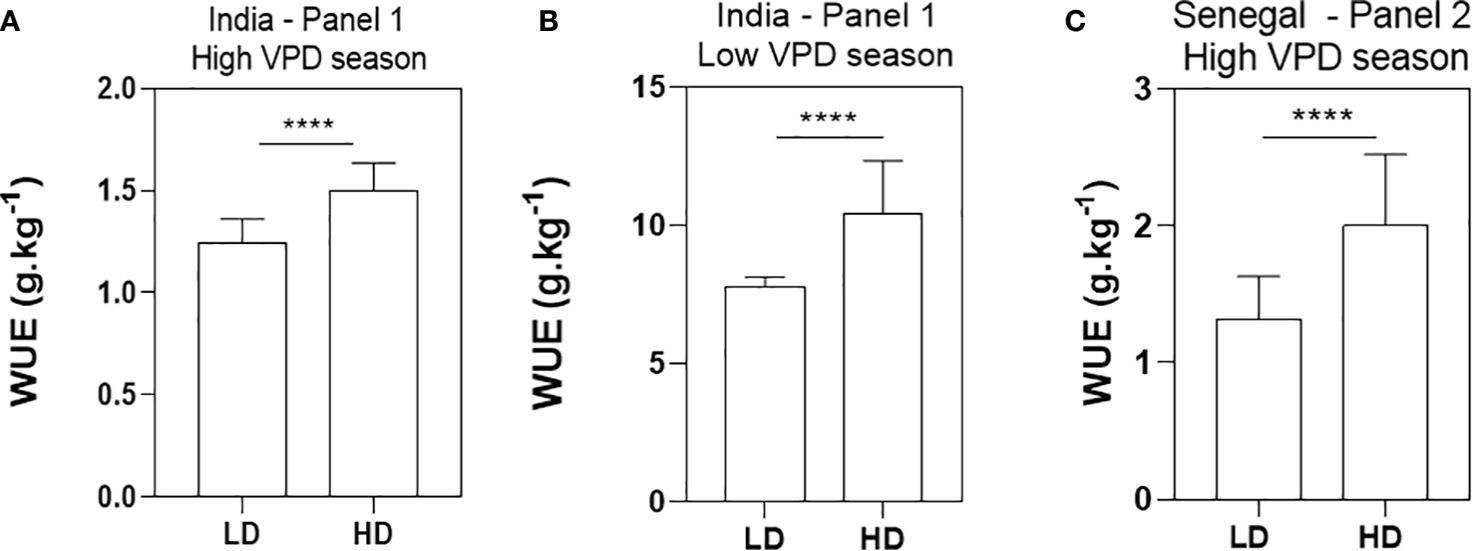
Water use efficiency (WUE) in grams of biomass per kilograms of water use in the low density (LD) and high density (HD) treatments. WUE was measured in 20 genotypes in the lysimeter platform in India in 2018 in both the dry **(A)** and rainy **(B)** seasons, and in 30 genotypes tested in the Senegal lysimeter platform during the 2021 post rainy season **(C)**. Data are means and standard deviation of 20 genotypic means in India **(A, B)** and 30 genotypic means in Senegal **(C)**. Genotypic mean values were the average of 4 replicated plots in India and 3 replicated plots in Senegal, for each genotype-by-density combination. ****p-value < 0.0001.

In all trials, the HD treatment increased WUE significantly. In the panel 1 in India, average WUE was 1.25 g biomass kg^-1^ water transpired in LD and 1.50 g biomass kg^-1^ water transpired in HD for the summer season trial (**Figure 5A**), and 7.80 g biomass kg^-1^ water transpired in LD and 10.41 g biomass kg^-1^ water transpired in HD for the rainy season trial (**Figure 5B**). However, a two-way ANOVA showed no significant genetic nor genotype-by-density interaction effects. Only the density effect was strongly driving the variation (Two-way ANOVA, p-value<0.0001). The same results were found in the high VPD season trial in Senegal for genotype panel 2. There, the HD treatment also increased WUE significantly. The average WUE for LD was 1.32 g biomass kg^-1^ water transpired versus 2 g biomass kg^-1^ water transpired for the HD treatment (**Figure 5C**). A two-way ANOVA showed a slightly significant genotypic effect (p-value = 0.02), and a much stronger density effect (two-way ANOVA, p-value<0.0001), but no genotype-by-density interaction effect. Density more than genotype effect drove the WUE variation (Wald statistic values: F_density_ = 50.9, F_genotype_ = 1.7).

### Soil water moisture is higher in the deepest part of the cores

**Figure 6:**
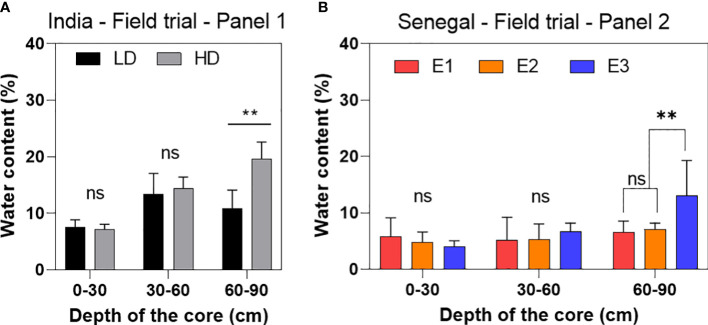
Percentage of soil moisture measured in three soil horizon profiles (0-30, 30-60 and 60-90cm) in the two density treatment (low density LD and high density HD) tested in India **(A)** and the three different density tested in Senegal **(B)** (E1, 3.2 plants/m^2^ - E2, 6.4 plants/m^2^ – E3, 12.8 plants/m^2^). Results were obtained through soil cores performed in the field immediately post-harvest. Means are average plus standard deviation of data collected in three plots for each of the density treatments. ns, non-significant. *p-value < 0.05.

From the 2018 field trial in India, water content differences between high and low-density treatments were found for the cores sampled between 60 and 90cm. Soil moisture in this part of the profile was 19.57% in HD, higher than the 10.78% moisture found in the LD treatment (t-test, p-value= 0.02). The upper parts (0-30 and 30-60cm) showed not significantly difference between density treatments (**Figure 6A**). In the 2020 field trial in Senegal, the soil moisture measured in the first two profiles (i.e. 0-30 an 30-60cm) showed no significant differences between the three densities. In the deepest part (60-90cm), although differences were not significant, we observe the same tendency as in the trial in India of a higher soil moisture in the deepest part and for the highest density (D3) with 7.9% against 5.9% and 5.7% for D1 and D2 respectively (**Figure 6B**).

## Discussion

The main results of the paper were: (i) the higher density increased biomass and/or grain yield in high VPD seasons; (ii) there was no or very limited genotype-by-density interaction in the response to density, indicating no genotypic variation in the response to density; (iii) The positive response to density was limited to seasons with no light limitation (iv) Higher density decreased VPD in the canopy and increased WUE in all cultivars.

The response to higher sowing density in 2017 and 2018 field trials in India and in the 2019 field trial in Senegal was positive for a large majority of the genotypes tested. These trials were conducted during seasons characterized by high evaporative demand. These results indicate that intensifying pearl millet productivity should be possible using existing cultivars, and that this recommendation should target area with expected high evaporative demand. To our knowledge, this is the first report of a positive response to density in pearl millet. It is consistent with the positive response to density found earlier in maize (Mansfield & Mumm, 2014), or sunflower (Echarte et al., 2020), where the positive response to increased density was also associated with a high intercepted radiation.

Although there was a positive response to density, we showed no genotype-by-density interaction, indicating that there was no genotypic variation in the degree of response to density. These results indicated that, at least according to the response of the 50 cultivars tested in this work, there seemed to be no avenue to breed for density tolerance in pearl millet. This contrast with the earlier work in maize where they found genotypic variation in the response of hybrids to an increased density (Mansfield & Mumm, 2014). This also contrast with a recent work in sorghum where the authors found significant genotype-by-density interaction indicative of genotypic differences in the response to density (Pilloni et al., 2022, unpublished). The reasons for this lack of variation is unclear but could involve architecture traits related to light penetration in the canopy, as recently shown in maize (Perez et al., 2019). It could be that the high tillering ability of pearl millet and its usually narrow and droopy leave pattern may have limited the genotypic variation for light penetration, at least in the sets of genotypes that were considered. More work would be needed to search for genotypic variation in pearl millet for traits that have been found to influence the response to an increased density in other crops (Mansfield & Mumm, 2014).

An environmental effect also altered the cultivar response to density, since the response to density in Senegal was positive in 2019 and negative in 2020. This is consistent with earlier report stating the importance of environmental conditions in the choice of an optimal density of planting in other crops (Reddy et al., 2009; Guzman et al., 2019). Here, the 2020 season largely differed from 2019 in the number of days of rain that occurred during the trial, leading to more days with less light available for the crop in 2020. This was also supported by data collected from a meteorological station located 25 km south of the CNRA Bambey station (Roupsard et al., 2018) during 2019 and 2020 where the average radiation during the 2019 crop cycle was 334.3 W.m².s^-1^ while the 2020 season was characterized by an average radiation of 304.2 W.m².s^-1^. This relation between the number of rainy days and the radiation reaching the ground is also supported by the literature (Az-Torres, 2017). This limitation may explain that plants grown in high density were experiencing a competition for light as this resource was less abundant in 2020, and would explain the negative effect of higher densities in that season. Therefore, the results from this work tell us that an intensification of pearl millet production is possible in Senegal and in semi-arid regions of India, and that this recommendation should focus on areas where the evaporative demand is high and where there is no light limitation.

Our results from the lysimeter also showed the positive density effect on the water use efficiency in all the three trials. However, as in the field trials, no genotypes-by-density interactions were found, except for a slight interaction in the Senegal trial, indicating that no genotypes had any stronger WUE response to density than any other did. The lower VPD we measured within the high density canopy in the lysimeter trials likely explained the increase in WUE (Ryan et al., 2016; Jauregui et al., 2018) as there is a negative relationship between WUE and VPD (Hatfield & Dold, 2019; T. R. Sinclair et al., 1984). The increase in LAI could explain this lowering of the VPD in the canopies (Gautier et al., 1999; Hu et al., 2008; Yang et al., 2014). This would imply that a substantial part of the incident light is able to penetrate inside the canopy and reaches lower level leaves. The fact there was no genotypic variation in the WUE response to density (no genotype-by-density interaction effect) suggest that light penetration would have been similar in all cultivars. We may speculate that, if the hypothesis is correct that light penetration inside the canopy helps decreasing canopy VPD and increasing WUE, genotypic variation in the response to sowing density in pearl millet could be found by in cultivars with canopy architecture differences, using traits identified earlier in maize as a benchmark (Mansfield and Munns, 2013; Perez et al., 2019).

**Figure 7:**
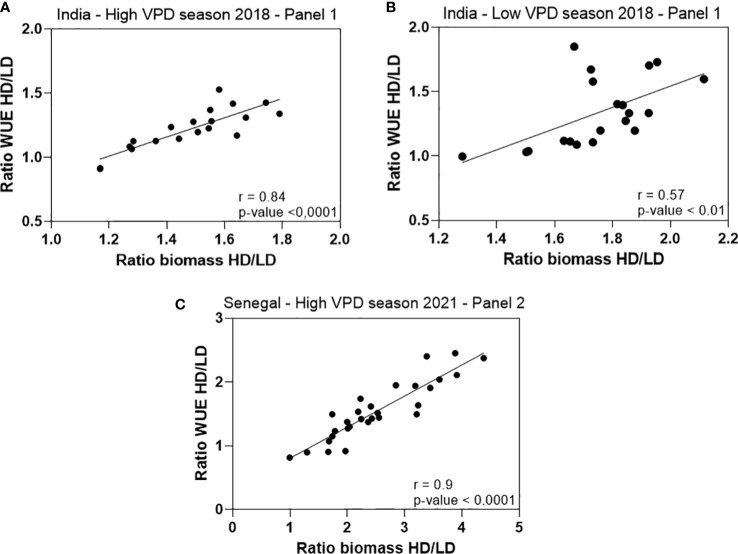
Correlation between the ratios of the WUE measured under high density to low density (WUE HD/WUE LD) as a function of the ratio of the biomass measured under high density to low density (Biomass HD/Biomass LD), using the data from the lysimeter trials carried out in India in 2018 and Senegal in 2021. Data shows positive and significant relation in both high VPD seasons in India (r = 0.84, p-value< 0.0001) **(A)** and Senegal (r = 0.9, p-value< 0.0001) **(B)** and also in the low VPD season in India (r = 0.57, p-value< 0.01) **(C)**. Ratios were calculated from the genotypic means of WUE and biomass in each of the density treatments.

The VPD measurement also showed that differences in VPD occurred only above a certain air VPD threshold. Data from the field in 2020, suggests a VPD threshold around 2kPa below which the benefit of high density was lost. Therefore, we propose that in dry and hot condition with no light limitation, there is a comparative advantage for the crop to be planted under high density, which would then create a microclimate with lower VPD within the canopy that eventually contribute to increasing WUE. On the contrary, in seasons with lower light availability, leaves would be competing for light, letting little or no light reach the depth of the canopy where VPD is milder. These assumptions are comforted by the fact that an increase in biomass was more positively related to an increase in WUE during the high VPD season in India and Senegal than during the low VPD season in India. Indeed, the ratio of the WUE obtained in HD to the one obtained in LD plotted against a similar ratio for biomass (these ratios representing a change in both WUE and biomass caused by the increase in density) showed a stronger correlation and significance in the high VPD season (r = 0.84, p-value< 0.0001 in India, r = 0.9, p-value< 0.0001 in Senegal) than in the low VPD one in India (r = 0.57, p-value< 0.01) (**Figure 7**). These relations are in line with our interpretation that the higher WUE would also have more than compensated the higher water demand caused by the higher LAI. Other report also show an increase in WUE under higher sowing density (Echarte et al., 2020), although this report made no interpretation in relation to a possible VPD effect in these changes. This is also consistent with our soil cores measurements, both in Senegal and India, where soil moisture was higher in the 60-90cm layer under high density than under low density. Milder microclimate and the better WUE in higher plant stands could have explained part of these differences. While the higher soil moisture at depth in the high-density treatments remains in part unexplained, it opens the door for root system investigation in response to increased density.

## Conclusion

This work showed a positive effect of increasing sowing density on the biomass and grain yield of pearl millet, in different location and genotypes. The benefit of higher density was maximum when the evaporative demand was high, suggesting an avenue to avoid atmospheric drought stresses, to the benefit of the water use efficiency of the crop and then of the yield. The strong environmental influence on the response of varieties already in use by farmers suggest the density have to be adapted according to areas and associated climate.

## Data availability statement

The raw data supporting the conclusions of this article will be made available by the authors, without undue reservation.

## Author contributions

RP, AK, RB, CF carried out the experimental work, VV, AF, RP, and JK designed the experiments, RP, VV, and AF analysed the data, RP, VV and AF wrote the paper. All authors contributed to the article and approved the submitted version.

## Supplementary material

Supplementary Figure 1 Schematic of the sowing pattern in both high and low density in the fields in India **(A)** and the three densities in the fields in Senegal **(B)**.

Supplementary Figure 2 Schematic of a replication in both high and low density, which consisted in four tubes in the two lysimetric platform in both India and Senegal **(A)**. Schematic representing an overview of a lysimetric trial with several replications set up on the platform **(B)**.
