# Supplementary figures and images for: Higher sowing density of pearl millet increases productivity and water use efficiency in high evaporative demand seasons
Source: Front Plant Sci. 2022 Dec 8;13:1035181. doi: 10.3389/fpls.2022.1035181 (PMC9773418; doi:10.3389/fpls.2022.1035181)

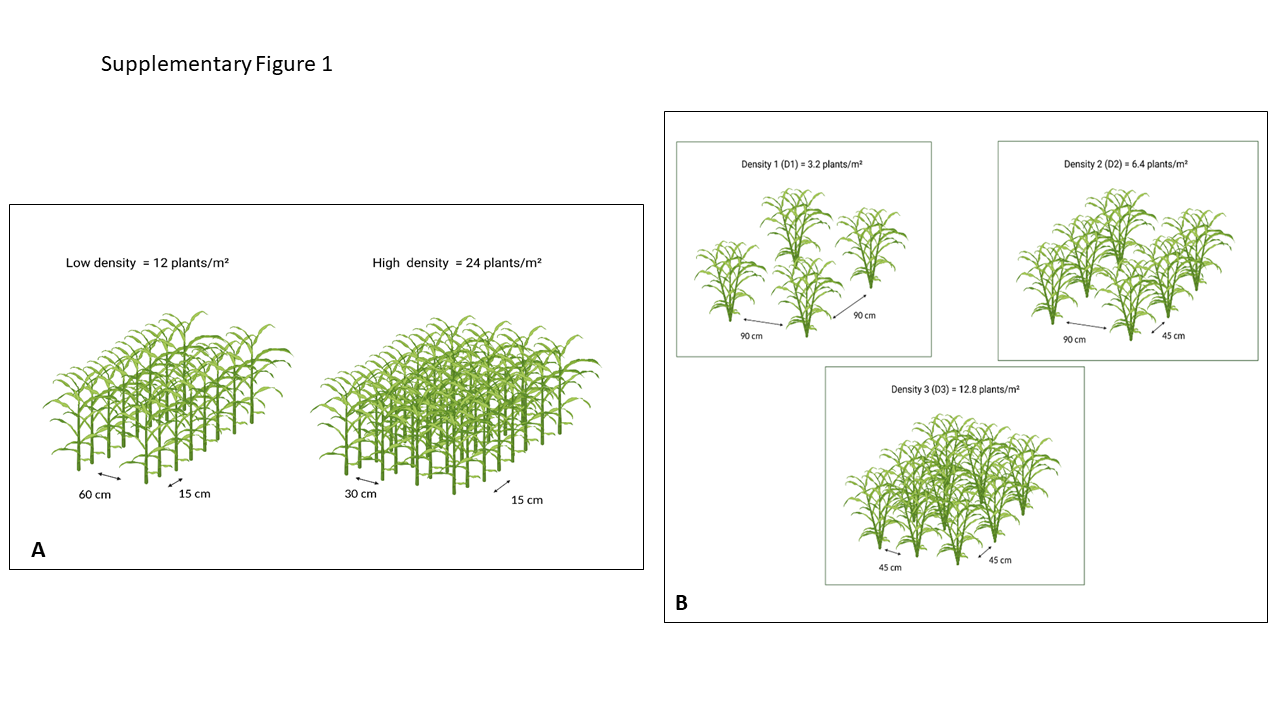

Supplement: Supplementary Figure 1 — Schematic of the sowing pattern in both high and low density in the fields in India (A) and the three densities in the fields in Senegal (B). [file Image_1.tif]

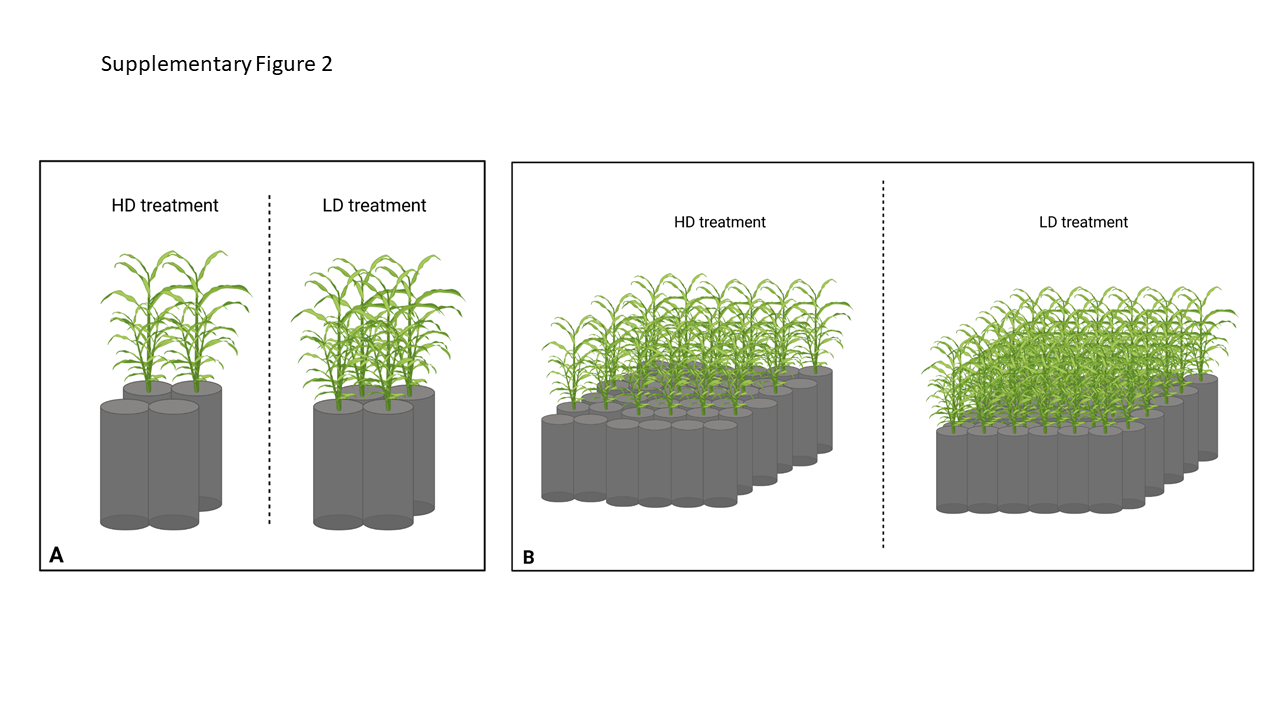

Supplement: Supplementary Figure 2 — Schematic of a replication in both high and low density, which consisted in four tubes in the two lysimetric platform in both India and Senegal (A). Schematic representing an overview of a lysimetric trial with several replications set up on the platform (B). [file Image_2.tif]
