# Supplementary material for: Higher sowing density of pearl millet increases productivity and water use efficiency in high evaporative demand seasons
Source: Front Plant Sci. 2022 Dec 8;13:1035181. doi: 10.3389/fpls.2022.1035181 (PMC9773418; doi:10.3389/fpls.2022.1035181)
Supplement: Supplementary file 3 [file DataSheet_1.docx]

Supplementary Table 1: Grain and biomass yield in both high and low density from 2017 and 2018 field trials in India. Values are expressed in ton per ha and are means of three replications.

|  | 2017 dry sesaon (tons/ha) | | 2018 dry season (tons/ha) | |
| --- | --- | --- | --- | --- |
| Genotypes | LD grain yield | HD grain yield | LD biomass yield | HD biomass yield |
| HT 416628 | 3,59 | 3,73 | 5,66 | 5,92 |
| GK 1183 | 3,92 | 3,85 | 4,92 | 6,89 |
| HYMH 5 | 3,87 | 3,98 | 5,52 | 7,25 |
| Bio 549 | 2,49 | 4,00 | 5,64 | 7,88 |
| KH 3022 | 3,03 | 4,11 | 6,08 | 6,83 |
| 86 M 86 | 3,34 | 4,14 | 5,57 | 6,43 |
| NU 399 | 4,00 | 4,19 | 6,56 | 7,37 |
| Super Boss | 3,52 | 4,57 | 5,06 | 7,24 |
| NU409 | 4,04 | 4,71 | 5,31 | 6,89 |
| 9444 | 3,42 | 4,72 | 4,71 | 7,17 |
| GK 1235 | 3,95 | 4,76 | 5,73 | 6,39 |
| GK1207 | 3,82 | 4,78 | 5,55 | 5,11 |
| 86 M 88 | 3,64 | 4,84 | 5,36 | 5,73 |
| Bio 451 | 4,54 | 4,84 | 6,40 | 8,53 |
| NBH 5863 | 4,26 | 4,90 | 6,77 | 7,40 |
| HYMH 8 | 3,71 | 4,97 | 5,45 | 6,92 |
| BLMPH 105 | 5,00 | 5,01 | 5,78 | 7,04 |
| JKBH 1490 | 4,48 | 5,12 | 6,36 | 7,09 |
| JKBH 1352 | 5,01 | 5,32 | 6,68 | 5,69 |
| APH 43 | 4,57 | 5,71 | 5,85 | 7,06 |

Supplementary Table 2: Grain yield in the three densities set up in 2019 and 2020 rainy seasons field trials in Senegal. Values are expressed in ton per ha and are means of three replications.

|  | 2019 rainy season | | | 2020 rainy season | | |
| --- | --- | --- | --- | --- | --- | --- |
| Genotypes | D1 grain yield | D2 grain yield | D3 grain yield | D1 grain yield | D2 grain yield | D3 grain yield |
| CHAKTI | 1,80 | 1,66 | 1,69 | 0,92 | 0,65 | 0,52 |
| GB8735 | 1,59 | 1,55 | 1,66 | 0,96 | 1,06 | 0,81 |
| SL423 | 0,71 | 0,78 | 0,79 | 1,68 | 1,59 | 1,09 |
| ICMH177111 | 2,13 | 1,19 | 1,31 | 2,43 | 2,51 | 1,15 |
| ICMHIS14006 | 2,93 | 2,71 | 3,07 | 1,67 | 1,57 | 1,86 |
| ICMHIS14007 | 2,70 | 1,81 | 3,76 | 1,87 | 1,79 | 1,95 |
| ICMP177001 | 2,10 | 1,37 | 2,28 | 0,82 | 0,84 | 0,98 |
| ICMP177002 | 0,80 | 0,81 | 1,44 | 0,81 | 0,57 | 0,67 |
| ICMV147141 | 0,57 | 1,02 | 1,64 | 1,80 | 2,21 | 1,82 |
| ICMV147142 | 2,78 | 3,04 | 3,54 | 1,76 | 1,65 | 1,45 |
| ICMC147143 | 1,59 | 1,46 | 2,62 | 0,53 | 0,60 | 0,50 |
| ICMV147144 | 0,72 | 0,33 | 0,48 | 1,11 | 1,06 | 0,99 |
| ICMV167001 | 0,90 | 0,96 | 1,32 | 0,98 | 0,74 | 0,73 |
| ICMV167002 | 0,98 | 1,21 | 1,25 | 1,28 | 1,29 | 1,32 |
| ICMV167003 | 1,09 | 1,73 | 1,75 | 1,14 | 1,59 | 1,11 |
| ICMV167004 | 1,36 | 2,33 | 3,47 | 1,58 | 1,62 | 1,47 |
| ICMV167005 | 0,64 | 1,83 | 2,47 | 1,73 | 1,81 | 1,68 |
| ICMV167006 | 0,69 | 1,26 | 1,21 | 1,88 | 1,67 | 1,42 |
| ICMV167012 | 1,26 | 1,55 | 2,07 | 2,10 | 1,90 | 1,35 |
| PEO8043 | 1,66 | 1,72 | 1,83 | 2,33 | 1,30 | 1,08 |
| ICMVIS89305 | 1,87 | 2,59 | 3,73 | 1,89 | 2,41 | 1,93 |
| ICMVIS92222 | 0,84 | 1,05 | 2,04 | 1,77 | 1,39 | 1,50 |
| ICMVIS94206 | 1,72 | 2,03 | 1,29 | 1,18 | 1,60 | 1,93 |
| ICMVIS99001 | 1,05 | 1,75 | 1,97 | 1,98 | 2,03 | 1,43 |
| SOSAT-C88 | 1,19 | 1,24 | 3,55 | 1,50 | 1,50 | 1,03 |
| LCICMV-4 | 1,70 | 1,69 | 1,86 | 2,27 | 2,29 | 1,63 |
| ICMP187092 | 2,02 | 1,66 | 2,61 | 0,94 | 0,88 | 0,93 |
| ICMP187093 | 1,20 | 1,32 | 1,65 | 1,83 | 1,45 | 1,34 |
| ICMB177002 | 0,63 | 0,94 | 2,11 | 2,46 | 2,46 | 2,44 |
| ICMR08888 | 2,19 | 1,99 | 2,48 | 2,56 | 1,97 | 1,83 |
